# Supplementary material for: Organic hydrogen peroxide-driven low charge potentials for high-performance lithium-oxygen batteries with carbon cathodes
Source: Nat Commun. 2017 Jun 6;8:15607. doi: 10.1038/ncomms15607 (PMC5467169; doi:10.1038/ncomms15607)
Supplement: Supplementary Information — Supplementary Figures [file ncomms15607-s1.pdf]

Supplementary Information

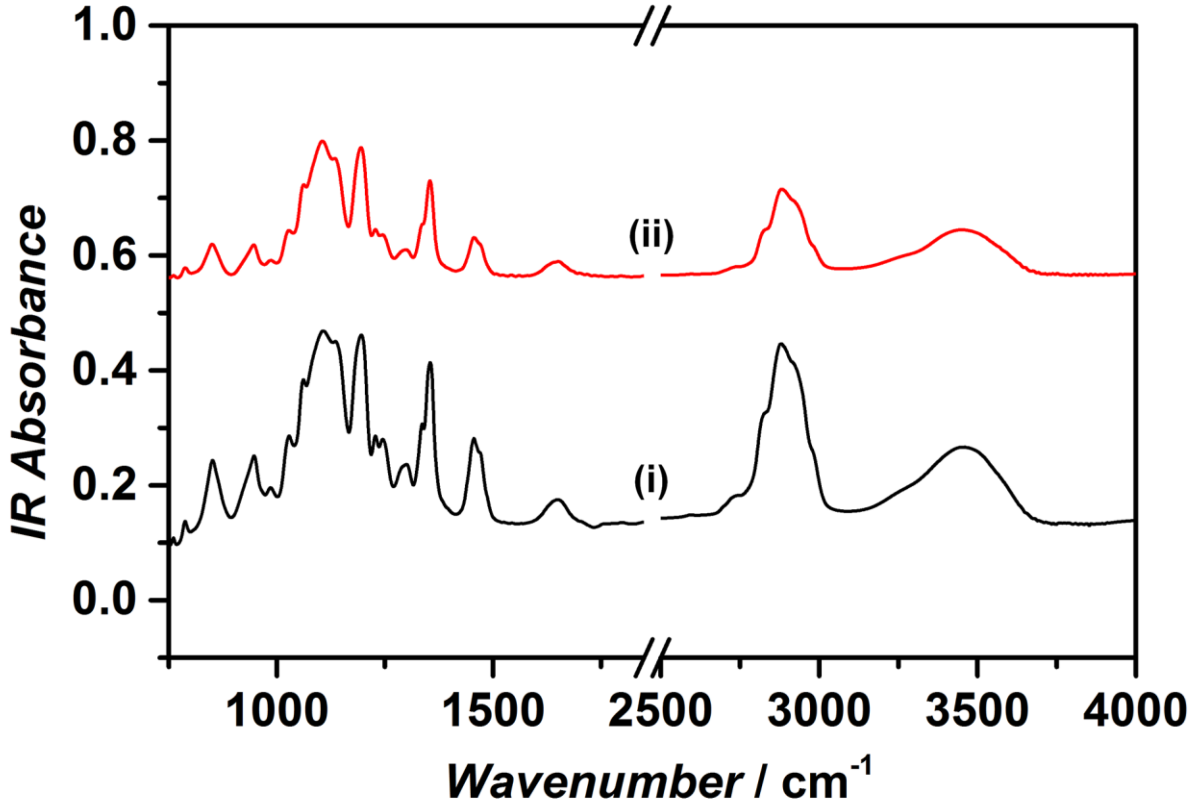

Supplementary Figure 1. Soluble species in electrolyte. FTIR spectra of (i) pristine G4-H_2O-H_2O_2 electrolyte and (ii) G4-H_2O-H_2O_2 electrolyte after charge, respectively.

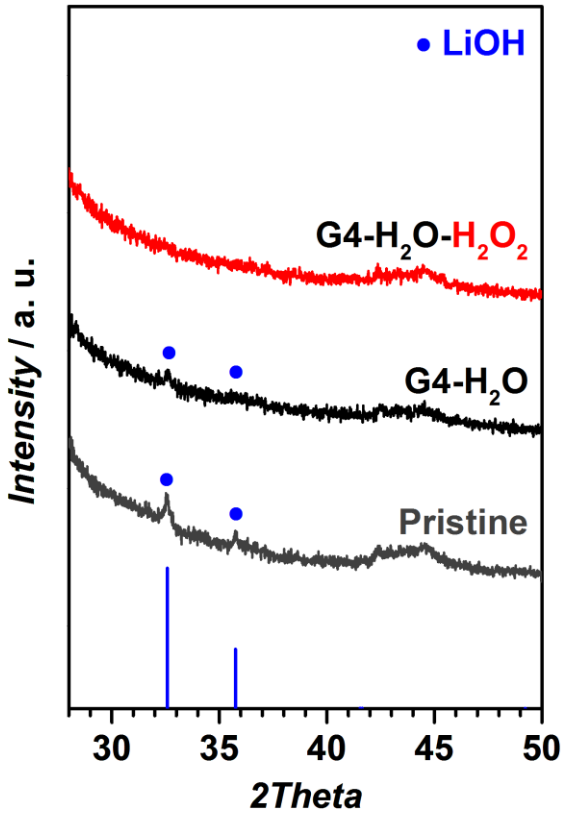

Supplementary Figure 2. XRD patterns of pristine LiOH-preloaded cathode (grey line) and cathodes after charge in G4-H_2O (black line) and G4-H_2O-H_2O_2 (red line) electrolytes.

The preloaded LiOH cannot be completely decomposed after charging in G4-H_2O electrolyte at high potential of > 4.3 V (Fig. 2a). While in G4-H_2O-H_2O_2 electrolyte, no diffraction peaks corresponding to the preloaded LiOH can be observed, suggesting the strong charge ability to decompose LiOH at the low potential of ~3.60 V. The important role of H_2O_2 in assisting LiOH decomposition can be concluded. The Li-O_2 pouch cell was used to avoid H_2O corrosion towards Li metal anode.

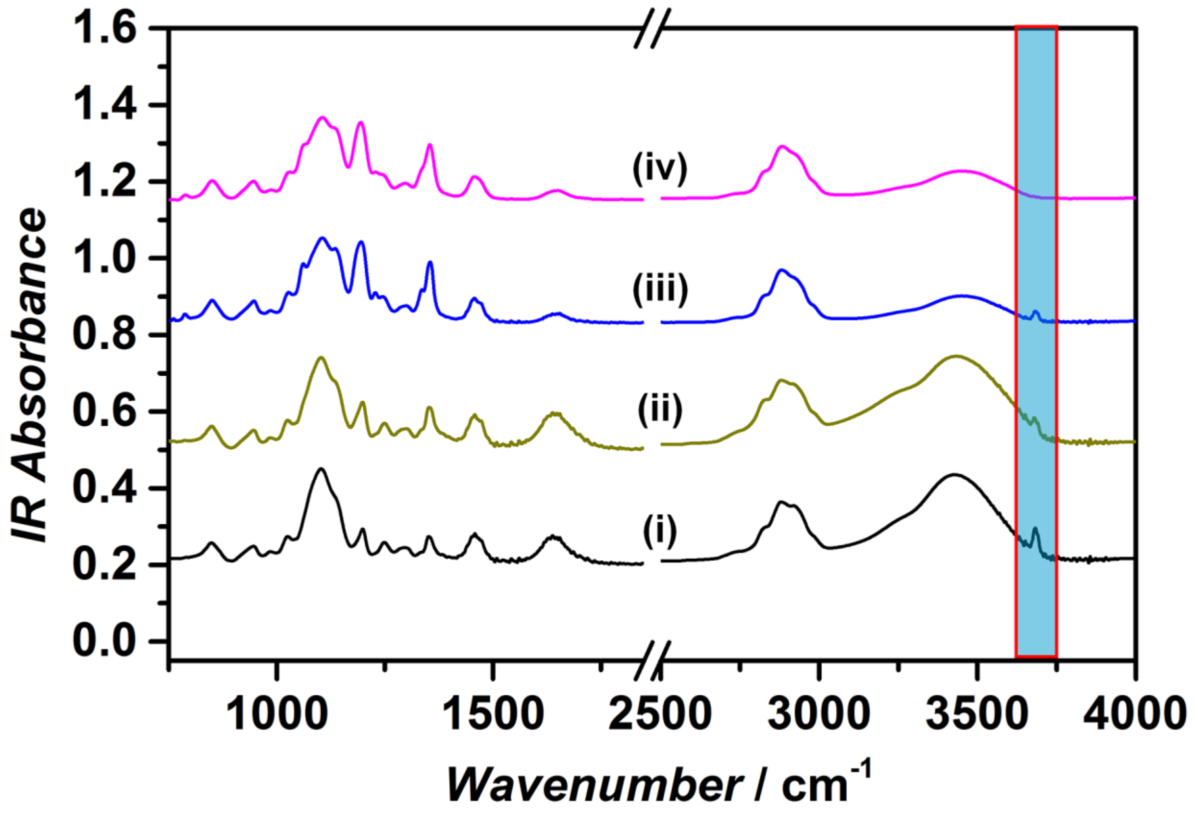

Supplementary Figure 3. Decomposition of liquid LiOH in electrolyte. FTIR spectra of (i) pristine G4-H_2O-$LiOH_{(l)}$ electrolyte, (ii) G4-H_2O-$LiOH_{(l)}$-H_2O_2 electrolyte, (iii) G4-H_2O-$LiOH_{(l)}$ electrolyte after charge and (iv) G4-H_2O-$LiOH_{(l)}$-H_2O_2 electrolyte after charge, respectively. The peak at ~3680 cm^{-1} corresponds to liquid LiOH in electrolyte.

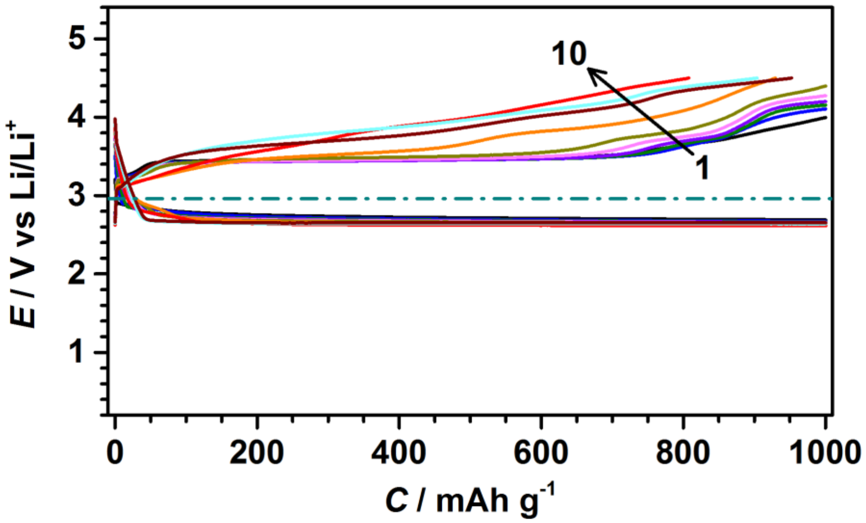

Supplementary Figure 4. Cycling performance of Li-O_2 pouch cell with LiSICON film and H_2O_2-containing electrolyte. The specific capacity is limited to 1000 mAh g^{-1}_{KB}. Current density: 100 mA g^{-1}_{KB}.

Although the charge overpotential is low in the initial cycles, the cycling stability is dissatisfactory, possibly due to the increased impedance of charge transfer through LiSICON film (Supplementary Fig. 5).

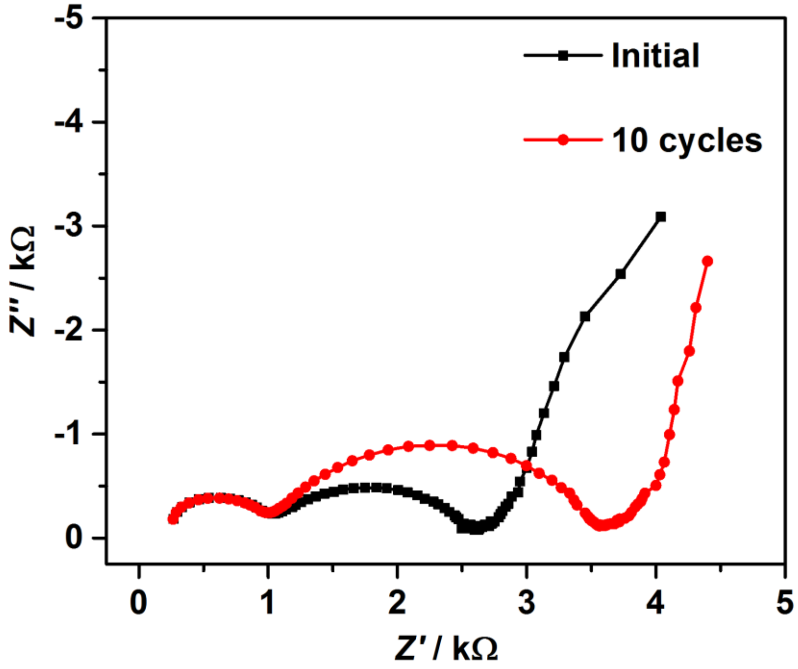

Supplementary Figure 5. Electrochemical impedance spectra of Li-O_2 pouch cell before and after cycles. Amplitude: 5 mV, Frequency: 10^{-2} - 10^6 Hz.

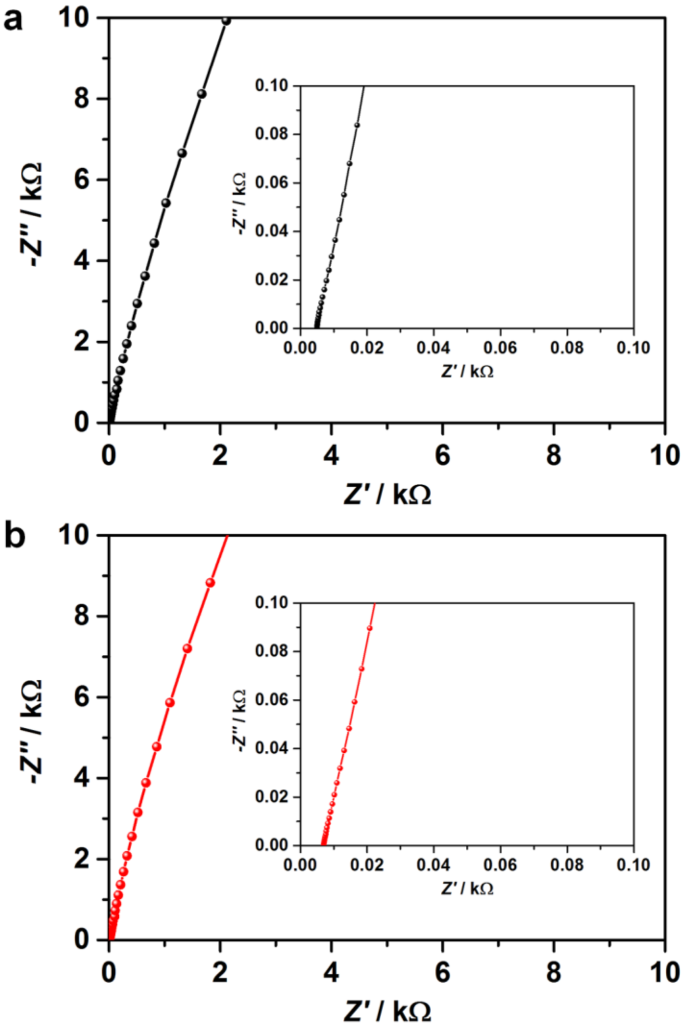

Supplementary Figure 6. Ion conductivity measurement. Nyquist plots of the (a) G4-based electrolyte and (b) G4-UH_2O_2 electrolyte with the cell structure of stainless steel (SS)/electrolyte/SS. Insets are the amplified figures.

From the Nyquist plots, the ion conductivity σ can be calculated via the equation $\sigma = d/(R_bS)$, where d is the thickness of the applied separator (20 μm), R_b is the resistance obtained from the Nyquist plot, and S is the surface area of the stainless steel (1.767 cm^2). The calculated ion conductivities are 2.0×10^{-3} S cm^{-1} for G4-based electrolyte and 1.8×10^{-3} S cm^{-1} for G4-

UH_2O_2 electrolyte, respectively. After introduction of UH_2O_2 into electrolyte, the Li^+ ion conductivity is still high and there is nearly no change of ion conductivity compared with G4-based electrolyte.

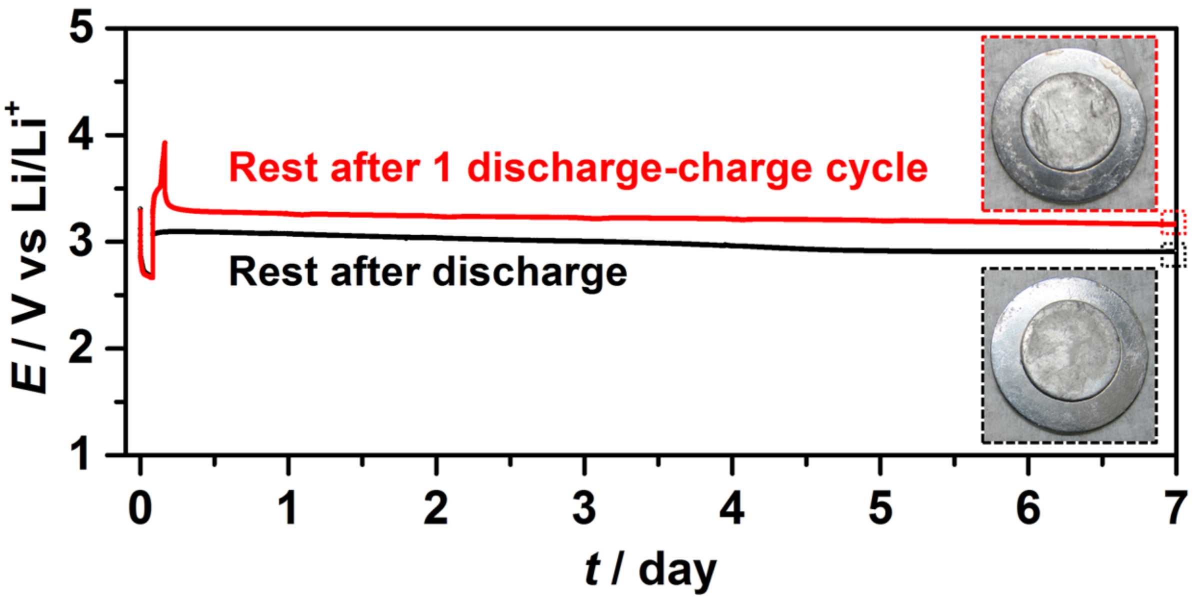

Supplementary Figure 7. Open circuit voltage (OCV) trends of Li-O_2 cells with UH_2O_2 additive in electrolyte after a discharge process and one discharge-charge cycle, respectively. Insets are the photos of Li metal anodes after rest.

The stability of UH_2O_2 additive is evaluated through a storage experiment. One cell was firstly discharged and then rested for 7 days. Another cell was firstly performed for one discharge-charge cycle and then rested for 7 days. The open circuit voltages (OCVs) were monitored (Supplementary Fig. 7). It can be seen that during these 7 days, the OCVs shows good stability and after rest, the Li metal anodes still keep uncontaminated by H_2O, the possible decomposition product of UH_2O_2. In addition, the Li-O_2 cell performs stable discharge-charge ability for 50 cycles (Fig. 7) and there is no obvious change of Li metal anode after cycles (Fig. 4). Therefore, the stability of the UH_2O_2 additive can be confirmed.

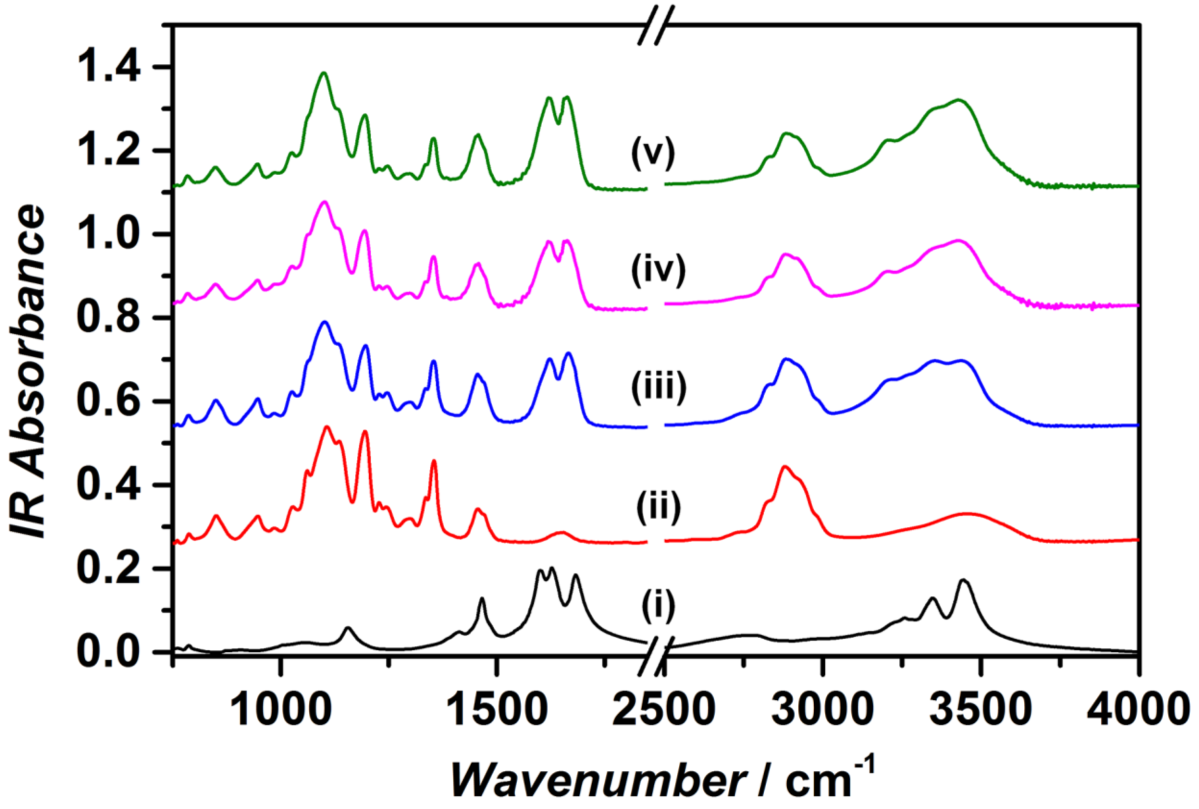

Supplementary Figure 8. Soluble species in G4-UH_2O_2 electrolyte. FTIR spectra of (i) pristine UH_2O_2 powder, (ii) G4-based electrolyte, (iii) G4-UH_2O_2 electrolyte, (iv) G4-UH_2O_2 electrolyte after discharge and (v) G4-UH_2O_2 electrolyte after recharge, respectively. In all the electrolytes, 1 M LiTFSI is dissolved.

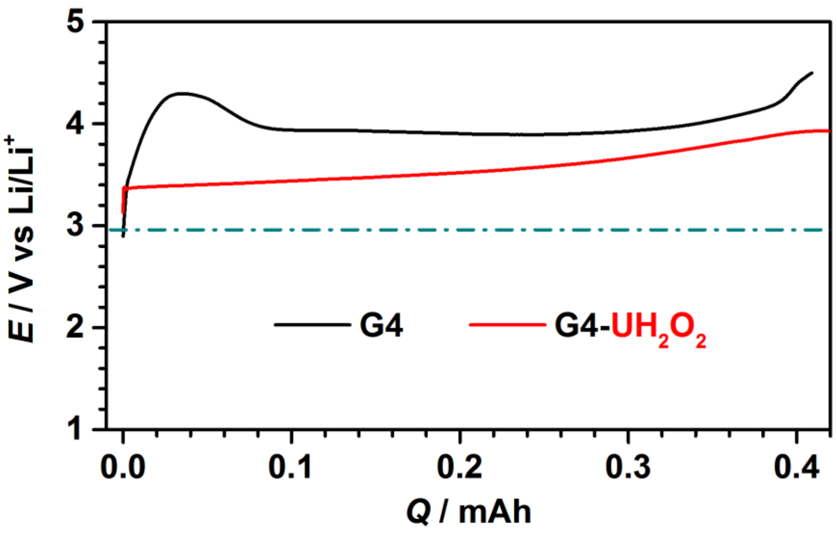

Supplementary Figure 9. The charge profile of Li-O_2 cell with Li_2O_2-preloaded KB cathode in the G4-based (black line) and G4-UH_2O_2 (red line) electrolyte. Current density: 100 mA g^{-1}.

In the G4-based electrolyte without any additive, the charge potential increases rapidly at the beginning and has to overcome a mountain at ~4.32 V. After that, the charge potential keeps at ~3.95 V for decomposing the preloaded commercial Li_2O_2. In contrast, in the G4-UH_2O_2 electrolyte, the charge barrier is well circumvented and the charge potential is reduced to the low value of ~3.48 V. The large promotion of charge ability for decomposing the preloaded Li_2O_2 due to the introduction of UH_2O_2 is emphasized. This higher charge efficiency may benefit the development of Li metal-free Li-O_2 battery, which has to use the Li_2O_2-preloaded cathode to provide Li ions.

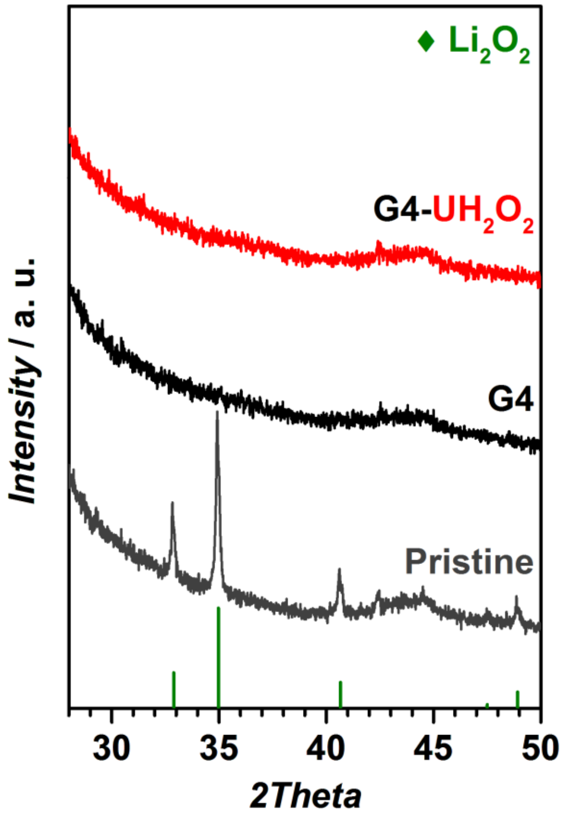

Supplementary Figure 10. XRD patterns of pristine Li_2O_2-preloaded cathode (grey line) and cathodes after charge in G4-based (black line) and G4-UH_2O_2 (red line) electrolytes.

Although the preloaded Li_2O_2 can be completely decomposed evidenced from the disappearance of diffraction peaks either in G4-based electrolyte or in the G4-UH_2O_2 electrolyte, the consumed energy in the G4-based electrolyte is much higher than that in the G4-UH_2O_2, suggested from the higher charge potential in Supplementary Fig. 9.

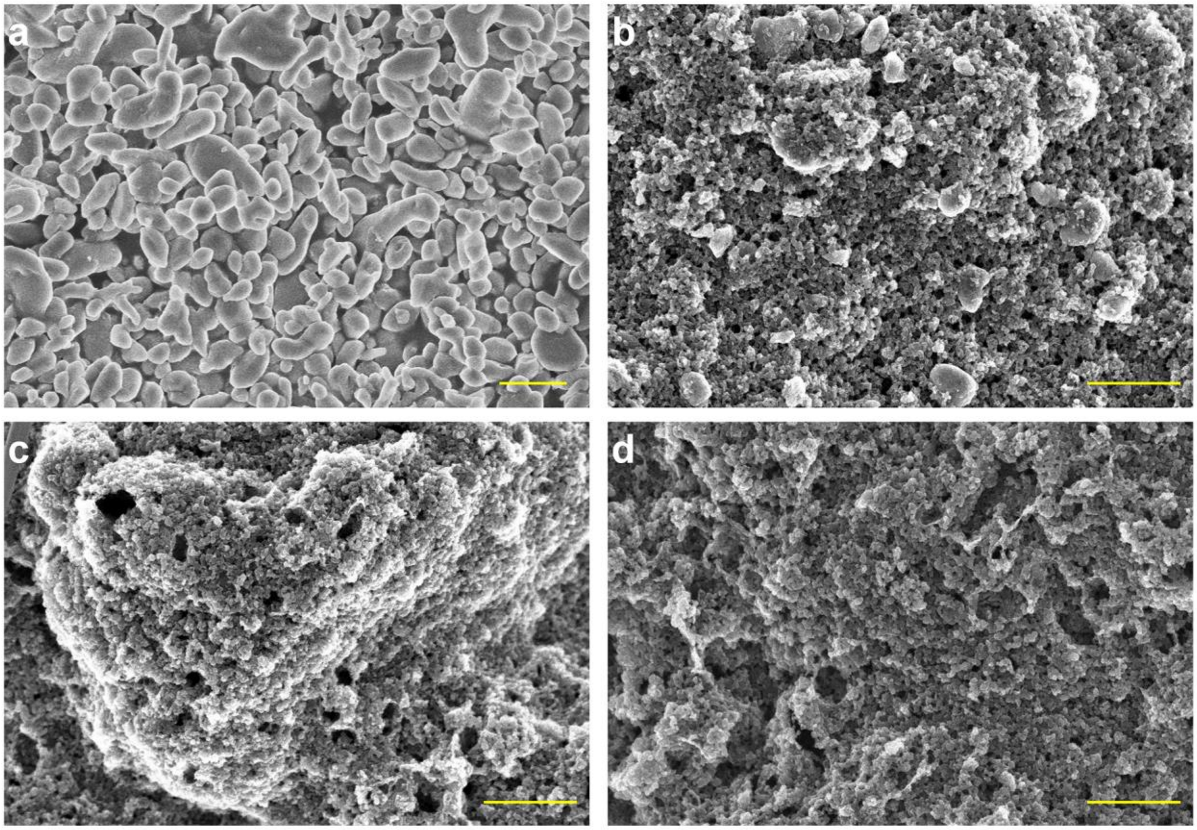

Supplementary Figure 11. Decomposition of preloaded Li_2O_2. SEM images of (a) the commercial Li_2O_2 particles, (b) the Li_2O_2-preloaded KB cathode and the charged Li_2O_2-preloaded KB cathodes in (c) G4-based and (d) G4-UH_2O_2 electrolytes, respectively. Scale bar: 1 μm.

The diameters of commercial Li_2O_2 particles are about 0.5 – 1 μm in Supplementary Fig. 11a. After preloading Li_2O_2 on KB, the mixture of Li_2O_2 and KB can be identified from SEM image in Supplementary Fig. 11b. After full charge in G4-based and G4-UH_2O_2 electrolytes, respectively, no Li_2O_2 particles can be observed in Supplementary Fig. 11c and d, leaving voids surrounded by KB particles. Combining the disappearance of Li_2O_2 diffraction peaks in XRD

patterns (Supplementary Fig. 10), it is can be confirmed that the preloaded Li_2O_2 can be completely decomposed either in G4-based electrolyte or in the G4-UH_2O_2 electrolyte.
